# Genomic abundance and transcriptional activity of diverse *gypsy* and *copia* long terminal repeat retrotransposons in three wild sunflower species

**DOI:** 10.1186/s12870-017-1223-z

**Published:** 2018-01-05

**Authors:** Fan Qiu, Mark C. Ungerer

**Affiliations:** Division of Biology, Kansas State University, Manhattan, KS 66506 USA

**Keywords:** Retrotransposons, *Gypsy*, *Copia*, Genomic abundance, Transcriptional activity, Sunflower

## Abstract

**Background:**

Long terminal repeat (LTR) retrotransposons are highly abundant in plant genomes and require transcriptional activity for their proliferative mode of replication. These sequences exist in plant genomes as diverse sublineages within the main element superfamilies (i.e., *gypsy* and *copia*). While transcriptional activity of these elements is increasingly recognized as a regular attribute of plant transcriptomes, it is currently unknown the extent to which different sublineages of these elements are transcriptionally active both within and across species. In the current report, we utilize next generation sequencing methods to examine genomic copy number abundance of diverse LTR retrotransposon sublineages and their corresponding levels of transcriptional activity in three diploid wild sunflower species, *Helianthus agrestis*, *H. carnosus* and *H. porteri.*

**Results:**

The diploid sunflower species under investigation differ in genome size 2.75-fold, with 2C values of 22.93 for *H. agrestis*, 12.31 for *H. carnosus* and 8.33 for *H. porteri*. The same diverse *gypsy* and *copia* sublineages of LTR retrotransposons were identified across species, but with *gypsy* sequences consistently more abundant than *copia* and with global *gypsy* sequence abundance positively correlated with nuclear genome size. Transcriptional activity was detected for multiple *copia* and *gypsy* sequences, with significantly higher activity levels detected for *copia* versus *gypsy*. Interestingly, of 11 elements identified as transcriptionally active, 5 exhibited detectable expression in all three species and 3 exhibited detectable expression in two species.

**Conclusions:**

Combined analyses of LTR retrotransposon genomic abundance and transcriptional activity across three sunflower species provides novel insights into genome size evolution and transposable element dynamics in this group. Despite considerable variation in nuclear genome size among species, relatively conserved patterns of LTR retrotransposon transcriptional activity were observed, with a highly overlapping set of *copia* and *gypsy* sequences observed to be transcriptionally active across species. A higher proportion of *copia* versus *gypsy* elements were found to be transcriptionally active and these sequences also were expressed at higher levels.

**Electronic supplementary material:**

The online version of this article (10.1186/s12870-017-1223-z) contains supplementary material, which is available to authorized users.

## Background

Long terminal repeat (LTR) retrotransposons are Class 1 transposable elements found in the nuclear genome of diverse forms of life [1]. These elements have been particularly successful at replicating in plants genomes [2, 3], where they often comprise a majority of nuclear DNA. *Gypsy* and *copia* elements represent the main autonomous superfamilies found in plants though additional nonautonomous forms (e.g., TRIMs, LARDs), also are recognized [4].

The ‘copy-and-paste’ mode of transposition of LTR retrotransposons requires transcriptional activation, with resulting RNA molecules serving as templates both for translation and reverse transcription [5]. Products of translation (e.g., gag, RT, INT) function in the multistep, autonomous life cycle whereas products of reverse transcription serve as the physical daughter copies that insert at new locations in the genome. This transcriptionally based, replicative mode of transposition enables LTR retrotransposons to achieve the exceptionally high copy numbers found within plant genomes.

It long has been suggested that LTR retrotransposons are transcriptionally inactive under normal conditions due to epigenetic suppression by the host genome. Natural selection should favor element suppression given potentially negative mutagenic effects of unchecked element activity. It is increasingly recognized, however, that transcriptional activity of LTR retrotransposons is more common than previously supposed, and may actually be a regular attribute of plant transcriptomes [6–12].

Though highly repetitive, LTR retrotransposons exist in plant genomes as diverse populations of sequences represented by different sublineages within the larger classification groups (i.e., superfamilies *gypsy* and *copia*). Genomic copy number abundance of elements within different sublineages can be highly variable, with elements from a small number of sublineages often comprising a disproportionately large fraction of the total genomic abundance [13–17]. The extent to which transcriptionally active LTR retrotransposons are representative of the total element diversity present in plant genomes is not well understood, nor is the relationship between expression level and genomic copy number abundance of specific sublineages. In the current report, we use NGS sequencing to investigate these relationships in three diploid wild *Helianthus* sunflower species by comparing genomic abundance and transcriptional activity levels for multiple sublineages of *gypsy* and *copia* LTR retrotransposons. We focus specifically on genomic abundance and transcriptional activity for a diverse panel of 40 full-length *gypsy* and 12 full-length *copia* elements previously shown to represent much of the LTR retrotransposon diversity in sunflowers [16–19]. Insertion age estimates of these elements in the common sunflower *Helianthus annuus* indicate that most have been active recently, with a mean insertion age of 0.7 million years in the *H. annuus* genome [19].

*Helianthus* species have been the focus of a range of investigations examining LTR retrotransposons and their contribution to genome evolution (reviewed in Giordani, et al. [20]) and transcriptional activity of these sequences has been reported for multiple species within the genus [7, 12, 17, 21–23]. Species under investigation in the current study include *H. agrestis*, the sunflower species with the largest estimated diploid genome size [17, 24], plus additional species *H. carnosus* and *H. porteri*. All three sunflower species exhibit relatively restricted geographic ranges in the SE United States, with populations of *H. agrestis* and *H. carnosus* largely restricted to the state of Florida [25, 26] and populations of *H. porteri* confined to granite outcrops in the southern Piedmont region of eastern Alabama, Georgia and South Carolina [25–27]. Despite similarly restricted ranges and geographic occurrences, these species occupy disparate locations in the *Helianthus* phylogeny, with *H. carnosus* belonging to a Southeastern perennial clade, *H. agrestis,* an annual, subtending a monophyletic group consisting of all perennial *Helianthus*, and with *H. porteri,* also an annual, positioned basal to all of *Helianthus* [26]. The phylogenetic placement of these species enable LTR retrotransposon genomic abundance and transcriptional activity patterns to be interpreted broadly across the sunflower genus.

## Methods

### Plant materials and growing conditions

Seeds of the plant species utilized in this study were acquired from the United States Department of Agriculture (USDA) National Plant Germplasm System (Table 1). Seeds were germinated on moist filter paper in Petri dishes and then transferred to 10 cm plastic pots containing a mixture (2:1) of Metro-mix 350: all purpose sand. Plants were grown in the Kansas State University greenhouse facility under a daily light cycle of 16 h light: 8 h dark, with supplemental lighting. Young, fully-expanded leaves were harvested from mature plants on the same date from three biological replicates per species, flash-frozen in liquid nitrogen, and stored at −70 °C prior to processing as described below.

**Table 1:** Genome size estimates for three *Helianthus* sunflower species

Species	Accession	Genome size [2C (SE)] accession mean	Genome size [2C (SE)] species mean
*H. agrestis*	673199	23.19 (0.30)	22.93 (0.32)
	468414	22.29 (0.64)	
	468416	23.31 (0.05)	
*H. carnosus*	649956	12.07 (0.64)	12.31 (0.24)
	664671	12.55 (0.01))	
*H. porteri*	649911	8.16 (0.18)	8.33 (0.12)
	649912	8.56 (0.06)	
	649918	8.27 (0.07)	

### Genome size estimation

Nuclear genome size was determined by flow cytometry using a Guava PCA-96 micro capillary system (Guava Technologies, Hayward, CA.). Sample preparation consisted of co-chopping with razor blades approximately 30 mg fresh leaf tissue each for the sample and an internal standard (*Secale cereal* cv. Dankovské) in a nucleus isolation buffer modified after Bino et al. [28] and consisting of 15 mM HEPES, 1 mM EDTA, 80 mM KCl, 20 mM NaCl, 300 mM sucrose, 0.5 mM spermine, 0.25 mM PVP-40, 15 mM β-mercaptoethanol and 0.2% Triton-X. Samples were then filtered through 30-μm nylon mesh, centrifuged to collect nuclei, and stained with propidium iodide solution (BioSure). Nuclear genome size estimates were obtained for 3 different populations of *H. agrestis* and *H. porteri* and for 2 different populations of *H. carnosus* (Table 1). Three biological replicates were assayed for each population, resulting in a total of 24 samples.

### DNA and RNA extraction, library construction and sequencing

DNA and RNA were extracted from leaf tissue using a DNeasy Plant Mini Kit (Qiagen, Valencia, CA) and TRIzol reagent (Invitrogen, Carlsbad, CA), respectively, following manufacturer instructions. Total RNA was purified to avoid any genomic DNA contamination using DNAse I and a RNeasy Mini Kit (Qiagen, Valencia, CA). One microgram of total DNA and RNA per sample was utilized for library preparation and sequencing on an Illumina HiSeq2500 platform, generating 2 × 100 bp paired-end reads for both datasets. Library preparation was performed following the Illumina TruSeq DNA PCR-free Library Prep Kit and the Illumina TruSeq RNA Sample Prep Kit v2 (Illumina Inc., San Diego, CA, USA). Average insert sizes were 470 bp and 260 bp for DNA and RNA libraries, respectively. Library construction and sequencing were performed at the University of Kansas Genome Sequencing Core Facility, Lawrence, KS, USA (http://gsc.drupal.ku.edu/).

Raw reads from DNA- and RNA-seq were trimmed using CLC Genomics Workbench v8.5.1 (Qiagen, Valencia, CA) to remove low quality sequences (PHRED scores <30) and sequences <80 bp and <40 bp, respectively. More stringent filtering of DNA-seq data was performed on account of the program RepeatExplorer [29] requiring input sequences ≥ 80 bp in length for analysis. After quality filtering, chloroplast- and mitochondrial-derived sequences were identified and removed by mapping reads against the complete chloroplast genome (GenBank accession number DQ383815) and complete mitochondrial genome (KF815390) of *Helianthus annuus*.

### Genomic repetitive fraction

The genomic repetitive fraction of each species was determined using a graph-based clustering approach developed by Novak et al. [29] and implemented in RepeatExplorer [30] on the Galaxy Server (http://www.repeatexplorer.org/). Briefly, approximately 3 M single end DNA-seq reads were randomly sampled from each biological replicate per species and clustered based on an all-by-all comparison of sequence similarity (≥ 90%) and sequence overlap (≥ 55%). Individual clusters were identified and counted toward the genomic repetitive fraction if they contained ≥0.01% of the starting number of sampled sequences (e.g., for 3 M sequences, minimum cluster size = 300 sequences). These parameter values represent default setting of RepeatExplorer.

### LTR-RT identification and global expression

Sequence clusters obtained from DNA-seq reads were identified as *gypsy* and *copia* LTR retrotransposons based on a similarity search, implemented in RepeatExplorer, of clustering output against a custom database consisting of 40 full-length *gypsy* and 12 full-length *copia* LTR retrotransposons derived from the *H. annuus* genome [18, 19]. These sequences represent a panel of diverse and variably abundant elements found in the genome of *H. annuus* [19], several additional *Helianthus* species [17], as well as in additional species in Asteraceae outside *Helianthus* [16]. These sequences are available as supplementary File S1 in Tetreault and Ungerer [17]. We followed the classification of sublineages in [17] for our LTR-RT identification and expression analysis.

Transcriptional activity was estimated by mapping the filtered RNA-seq reads to this same custom database using the CLC Genomics Workbench v8.5.1 (Qiagen, Valencia, CA) with sequence similarity ≥80%. Each mapped paired-end read was counted as one mapped fragment, as was each broken mapped read. The total mapped fragments of each sublineage were standardized by the average length of full-length elements within the focal *gypsy* and *copia* sublineages and by the total number of filtered reads (fragments per kilobase per million sequences, FPKM) to quantify expression level. Transcriptionally active elements were defined as those with FPKM values ≥1. Only uniquely mapped reads were utilized in transcriptional activity assays, though results were qualitatively similar when analyses were performed with multiply mapped reads.

### Correlation between genomic repetitive fraction and expression level

Pearson’s *r* was calculated to evaluate correlation between genomic repetitive fraction and expression level for sublineages within *gypsy* and *copia* superfamilies. To account for the evolutionary history of different sublineages, Phylogenetic Independent Contrasts (PICs) were conducted with CONTRAST of PHYLIP v3.695 [31, 32]. Maximum likelihood trees of *gypsy* and *copia* elements were constructed using MEGA v6.0 [33] based on amino acid sequences of the reverse transcriptase domain. The best evolutionary model of protein sequences was estimated by ProtTest v2.0 [34].

## Results

### Nuclear genome size, sequencing data and genomic repetitive fraction

Within species, estimates of nuclear genome size varied 22.29–23.31 for *H. agrestis*, 12.07–12.55 for *H. carnosus* and 8.16–8.56 for *H. porteri*. Differences in 2C values among populations of the same species were not significant based on the sampling performed (*H. agrestis*, *F* = 1.8792, *P* = 0.23; *H. carnosus*, *t* = 0.7403, *P* = 0.50; *H. poteri*, *F* = 3.1982, *P* = 0.11). Nuclear genome size varies 2.75-fold across species with mean estimates of 22.93 for *H. agrestis,* 12.31 for *H. carnosus* and 8.33 for *H. porteri* (Table 1).

The Illumina Hi-Seq platform generated from 34.0 M to 63.5 M, and from 35.8 M to 56.9 M raw reads for DNA- and RNA-seq, respectively, for the species under investigation (Table 1 and Additional file 1: Table S1). After trimming and quality filtering, the numbers of reads in the final datasets ranged, respectively, from 24.6 M to 48.0 M and from 32.6 M to 51.7 M. Read lengths were reduced from their original size of 101 bases to mean lengths of 99.6 bases for DNA-seq and 93.8 bases for RNA-seq (Additional file 1: Table S1).

Graph-based clustering of ~3 M DNA-seq reads per species implemented in RepeatExplorer [30] yielded genomic repetitive fraction estimates of 83.33 ± 0.02% for *H. agrestis,* 74.48 ± 0.04% for *H. carnosus*, and 74.78 ± 0.20% for *H. porteri* (Table 1). The repetitive fraction estimate for *H. agrestis* is similar to a previous report for that species (82.12 ± 0.15%) [17] and newly generated estimates for *H. carnosus* and *H. porteri* are at the upper end, but within the range, reported for other diploid *Helianthus* species (68.17% - 75.26%) [17].

### LTR-RT identification and abundance

**Fig. 1:**
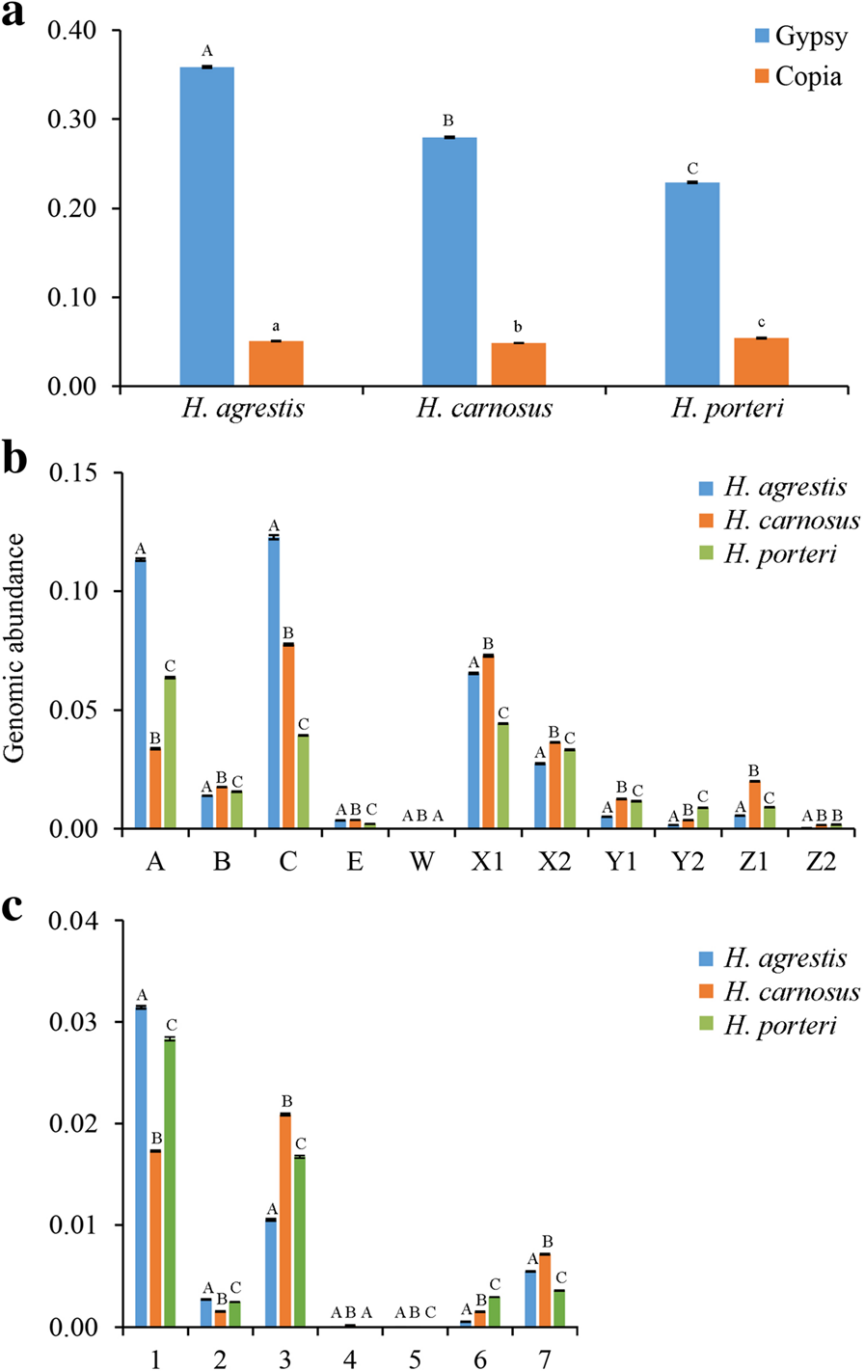
**a** Genomic abundance of *gypsy* and *copia* elements in three sunflower species based on similarity searches of graph-based clustering output to a reference panel of 52 full-length LTR retrotransposons. **b** and **c** Same information as in (**a**), but for different sublineages of *gypsy* (**b**) and *copia* (**c**). Error bars represent ±1 SE based on three biological replicates. Within a given element superfamily (panel **a**) or sublineage (panels **b** and **c**), histogram bars are labelled with different letter (A, B, C or a, b, c) when significant difference was determined (post-hoc Tukey test, *P* < 0.05)

To identify and quantify DNA-seq reads belonging to a diverse panel of annotated *gypsy* and *copia* LTR retrotransposons previously identified in the common sunflower (*H. annuus*) genome, sequence similarity searches of clustering output data from the graph-based clustering analysis were performed against a panel of 40 *gypsy* and 12 *copia* full length LTR-RT sequences reported originally in Buti et al. [18] and Staton et al. [19]. Between 22.92% ± 0.07 (*H. porteri*) and 35.85% ± 0.12 (*H. agrestis*) of sampled DNA-seq reads were identified as *gypsy*, and between 4.86% ± 0.01 (*H. carnosus*) and 5.41% ± 0.04 (*H. porteri*) were identified as *copia* (Fig. 1a), with *gypsy* sequences 4.2 to 7.1-fold more abundant than *copia* elements in these species genomes. While significant differences in abundance were observed among species for both read types (*gypsy*, *F* = 5.246, *P* < 10^−9^; *copia*, *F* = 118, *P* < 10^−4^, Fig. 1a), only *gypsy* sequence abundance exhibited a positive correlation with nuclear genome size (*r* = 0.998, *P* = 0.040 for *gypsy*; *r* = −0.494, *P* = 0.671 for *copia*, Additional file 1: Figure S1).

Sequence diversity is considerable within the LTR-RT reference panel utilized in this study, with the 40 full-length *gypsy* and 12 full-length *copia* sequences comprising multiple, well-supported sublineages based on phylogenetic analyses of the reverse transcriptase amino acid domain (Additional file 1: Figure S2). We thus examined genomic abundance of these various *gypsy* and *copia* sublineages in the context of this diversity. Considerable variation was observed within all three sunflower species in density of DNAseq reads assigned to different sublineages, with density of *gypsy* reads ranging from 3.97 × 10^−6^ to 0.12 for *H. agrestis*, from 8.56 × 10^−6^ to 0.08 for *H. carnosus* and from 3.56 × 10^−6^ to 0.06 for *H. porteri* and density of *copia* reads ranging from 5.67 × 10^−7^ to 0.03 for *H. agrestis*, from 2.78 × 10^−6^ to 0.02 for *H. carnosus* and from 1.16 × 10^−5^ to 0.03 for *H. porteri* (Fig. 1b and c). Within individual sublineages of *gypsy* and *copia*, significant differences in DNA-seq read abundance were observed among species in all instances, with the largest interspecific differences characterized by elevated abundance in *H. agrestis* for two sublineages of *gypsy* (sublineages A and C; Fig. 1b).

### Expression characteristics

**Fig. 2:**
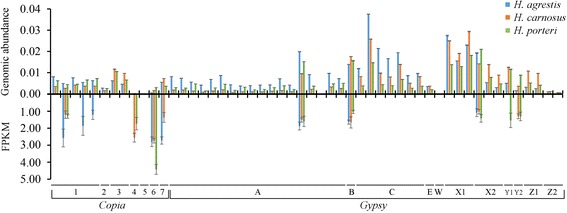
Genome abundance and expression level (FPKM) of 40 individual *gypsy* and 12 individual *copia* elements in three sunflower species. FPKM ≥1 was used as a cutoff for detectable expression. Error bars represent ±1 SE based on three biological replicates. Numerical and letter designations refer to different sublineages depicted in Additional file 1: Figure S2

**Fig. 3:**
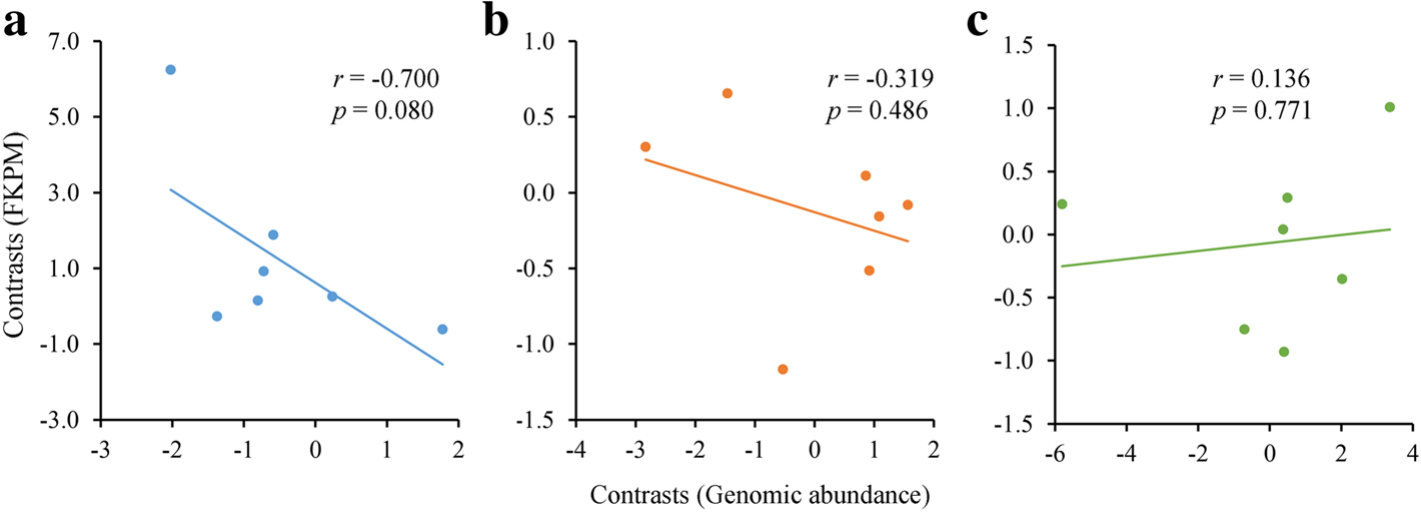
Phylogenetic independent contrasts between expression level (FPKM) and genomic abundance for *gypsy* and *copia* elements exhibiting FPKM ≥1 (see Fig. 2). Each data point represents an individual contrast, with Pearson’s *r* and *P* values calculated based on PIC analyses performed for *H. agrestis* (**a**), *H. carnosus* (**b**) and *H. porteri* (**c**)

Transcriptional activity of *copia* and *gypsy* sequences was determined by mapping RNAseq data to the reference panel of full-length LTR retrotransposons. Expression was detected for a total of 11 individual elements, consisting of 6 *copia* and 5 *gypsy* sequences (Fig. 2). Proportionally more *copia* (50%) versus *gypsy* (12.5%) elements were found to be expressed given the 1 FPKM threshold used for detection, and pooled across species, expression levels of *copia* elements were significantly higher than those for *gypsy* (*t* = 2.77; *P* = 0.011). Interestingly, expression of the same elements often was detected across multiple species, with 5 of 11 elements expressed in all three species, 3 of 11 expressed in 2 species and only the remaining 3 elements expressed in a single species. For elements determined to be active transcriptionally, regression analyses of FPKM and genomic abundance yielded nonsignificant results (Fig. 3a-c), though a negative trend was evident for *H. agrestis* and *H. carnosus.* Elements with the highest genome abundance estimates (e.g., those in *gypsy* sublineages C and X1) lacked transcriptional activity and the single *copia* element for which highest expression was detected in all three *Helianthus* species (i.e, *copia* sublineage 6) exhibited among the lowest genomic abundance estimates (Fig. 2).

## Discussion

Our understanding of LTR retrotransposon diversity and abundance in plant genomes has increased substantially in recent years on account of advances in genomics technology and the rapidly increased rate at which sequence data can be generated for both model and nonmodel species [13–17, 35]. For a majority of plant species examined thus far, both *gypsy* and *copia* superfamilies exist as diverse sublineages, with a small number of these sublineages often representing a disproportionately large fraction of the LTR retrotransposon and thus genomic component ([4], but see [35]). Growing evidence supported by unbiased RNAseq studies also indicates that LTR retrotransposons are expressed more commonly than previously supposed, despite longstanding belief that these sequences are transcriptionally inactive. It remains unknown, however, the extent to which diverse elements in plant genomes are expressed at detectable levels, and the precise relationship between genomic abundance of individual elements and likelihood, or level, of transcriptional activity. In the present work, we combined DNAseq and RNAseq datasets to investigate both genomic abundance and transcriptional activity levels of a diverse panel of LTR retrotransposons in three diploid wild sunflower species.

Analyses of DNAseq datasets indicate considerably higher genomic abundance of *gypsy* versus *copia* LTR retrotransposons in all three species, a result consistent with previous analyses of other sunflowers [16–19, 36], and other plant taxa [37–39], though biases of elevated *gypsy* abundances in plant genomes are not universal [40–43]. The three sunflower species under investigation here generally displayed similar patterns regarding which sublineages of *gypsy* and *copia* were most versus least abundant in the genome, though significant differences were detected among species in densities of reads within most sublineages. Highest read densities were observed in *H. agrestis,* with sequences represented by two *gypsy* sublineages comprising approximately 23% of all reads for that species. These observations are consistent with this species having the largest estimated nuclear genome size (Table 1), the highest genomic repetitive fraction (Table 2), and point to specific *gypsy* sublineages that likely contributed to genome expansion.

**Table 2:** Summary statistics of genomic data

Species	Accession used for sequencing	Raw paired-end reads (DNA-, RNA-seq) (M)	Trimmed paired-end reads (DNA-, RNA-seq) (M)	Repetitive fraction (%) (SE)
*H. agrestis*	673199	38.3–40.4, 40.0–44.0	28.6–29.5, 36.3–39.9	83.33 (0.02)
*H. carnosus*	649956	34.0–38.6, 48.5–56.9	24.6–28.3, 44.0–51.7	74.48 (0.04)
*H. porteri*	649918	42.4–63.5, 35.8–49.7	31.1–48.0, 32.6–45.2	74.78 (0.20)

*M* million, *SE* standard error

Transcriptional activity of both *gypsy* and *copia* LTR retrotransposons has been documented previously in both cultivated and wild sunflowers using targeted [12, 23, 44, 45] and unbiased [7, 21, 22] approaches. Low but detectable transcriptional activity was observed for multiple *gypsy* and *copia* elements in the current study, but with a higher proportion of *copia* elements found to be active transcriptionally and with these *copia* elements expressed at higher levels. These observations are consistent with the notion that elements that are more abundant in the genome (i.e., *gypsy*) are more likely to be targeted for silencing by the host genome and those at lower abundances more likely to escape such targeting. Expression variation of LTR retrotransposons has been observed in other plant species as well [46–48]. For example, in maize, retroelements identified as low-copy were found to be expressed whereas retroelements identified as abundant were not [49]. Such results are not universally observed, however, as positive (though non-significant) correlations also have been observed between retrotransposon copy numbers and transcription level across different elements [48].

An unanticipated finding of this study was the highly overlapping patterns across species of specific elements found to be transcriptionally active, with 5 of 11 expressed elements transcriptionally active in all three species and 3 of 11 found active in 2 species. This is an interesting result given the disparate locations of these species in the *Helianthus* phylogeny and estimated age of the genus at 1.7–8.2 Mya [50]. These results indicate that identical or highly similar elements retain the ability to be transcriptionally active and/or avoid host-specific targeting across the genus. It currently is unknown whether transcriptional activity of these LTR retrotransposons results in associated insertional activity, as posttranscriptional mechanisms of TE repression are not well understood in sunflowers. Posttranscriptional repression perhaps is suggested by patterns whereby individual elements exhibiting some of the highest expression levels also exhibit some of the lowest genomic abundances (e.g., *copia* 4 and *copia* 6). Such patterns should be interpreted with caution, however, as expression levels of all elements were generally low.

It is unlikely that the transcriptional activity patterns of LTR retrotransposons observed in this study have arisen from DNA contamination of RNA samples. While contamination issues are of clear concern when transcriptional activity of highly repetitive sequences are assayed, our results are counter to expectations if contamination was an issue. First, contamination would be expected to lead to positive correlations between element abundance levels and transcriptional activity levels. Individual elements exhibiting the highest abundance levels typically were found to be transcriptionally inactive. Second, for elements that were found to be active transcriptionally, positive relationships between FPKM and abundance were not observed, and in fact negative trends were apparent for two of the three species studied.

Epigenetic silencing mechanisms are known to function in host genomes to control retrotransposon proliferation and prevent potentially negative mutagenic effects of element transpositional activity [51]. These mechanisms can be achieved through both pre-transcriptional (such as RNA-directed DNA methylation; [51]) and post-transcriptional mechanism (such as RNA interference; [52, 53]). Studies investigating these silencing mechanisms mostly have focused on model plant systems such as *Arabidopsis thaliana*, rice and maize ([51]; and references therein). Genomic analyses of methylation and small RNA targeting in a comparative manner in sunflower may lead to a better understanding of the dynamics of host-TE interactions in the wild species under investigation here.

## Conclusions

While the three sunflower species under investigation in the current study differ markedly in genome size, patterns of LTR retrotransposon transcriptional activity were relatively conserved across species, with highly overlapping sets of *copia* and *gypsy* elements found to be transcriptionally active. For elements classified as transcriptionally active, negative but nonsignificant trend was observed between expression level (FPKM) and genomic abundance for two of three species. Transcriptional activity was not detected for individual elements exhibiting highest abundance. Additional experiments will be required to elucidate the mechanisms of transcriptional control and repression.

## Additional file

Additional file 1: Figure S1. Pearson’s correlation between density of reads and nuclear genome size for *gypsy* and *copia* elements. **Figure S2.** Phylogenetic relationship among different sublineages of (A) *gypsy* and (B) *copia* based on amino acid sequences of the reverse transcriptase domain (*gypsy*, 173 a.a.; *copia*, 250 a.a.) using maximum likelihood. Bootstrap scores are presented for internal branches with >50% support. **Table S1.** Summary statistics of DNA-seq and RNA-seq data. (PDF 488 kb)

## Abbreviations

FPKM: Fragments per kilobase per million sequences
INT: Integrase
LARDs: Large retrotransposon derivatives
LTR: Long terminal repeat
NGS: Next generation sequencing
PICs: Phylogenetic independent contrasts
RT: Reverse transcriptase
TRIM: Terminal-repeat retrotransposons in miniature

## References

[1] Eickbush TH, Malik HS. Origins and evolution of retrotransposons. In: Craig NL, Craigie R, Gellert M, Lambowitz AM, editors. Mobile DNA II. Washington, D.C.: ASM press; 2002. p. 1111–44.

[2] Grover CE, Wendel JF (2010). Recent insights into mechanisms of genome size change in plants. J Bot.

[3] Kumar A, Bennetzen JL (1999). Plant retrotransposons. Annu Rev Genet.

[4] Sabot F, Schulman AH (2006). Parasitism and the retrotransposon life cycle in plants: a hitchhiker's guide to the genome. Heredity.

[5] Schulman AH (2013). Retrotransposon replication in plants. Curr Opin Virol.

[6] Grandbastien MA (2015). LTR retrotransposons, handy hitchhikers of plant regulation and stress response. Biochim Biophys Acta.

[7] Kawakami T, Darby BJ, Ungerer MC (2014). Transcriptome resources for the perennial sunflower *Helianthus maximiliani* obtained from ecologically divergent populations. Mol Ecol Resour.

[8] Neumann P, Pozarkova D, Macas J (2003). Highly abundant pea LTR retrotransposon ogre is constitutively transcribed and partially spliced. Plant Mol Biol.

[9] Neumann P, Yan H, Jiang J (2007). The centromeric retrotransposons of rice are transcribed and differentially processed by RNA interference. Genetics.

[10] Parchman TL, Geist KS, Grahnen JA, Benkman CW, Buerkle CA (2010). Transcriptome sequencing in an ecologically important tree species: assembly, annotation, and marker discovery. BMC Genomics.

[11] Vicient CM, Jaaskelainen MJ, Kalendar R, Schulman AH (2001). Active retrotransposons are a common feature of grass genomes. Plant Physiol.

[12] Vukich M, Giordani T, Natali L, Cavallini A (2009). Copia and gypsy retrotransposons activity in sunflower (*Helianthus annuus* L.). BMC Plant Biol.

[13] Baucom RS, Estill JC, Chaparro C, Upshaw N, Jogi A, Deragon JM, Westerman RP, SanMiguel PJ, Bennetzen JL (2009). Exceptional diversity, non-random distribution, and rapid evolution of retroelements in the B73 maize genome. PLoS Genet.

[14] El Baidouri M, Panaud O (2013). Comparative genomic paleontology across plant kingdom reveals the dynamics of TE-driven genome evolution. Genome Biol Evol.

[15] Hawkins JS, Kim H, Nason JD, Wing RA, Wendel JF (2006). Differential lineage-specific amplification of transposable elements is responsible for genome size variation in Gossypium. Genome Res.

[16] Staton SE, Burke JM (2015). Evolutionary transitions in the Asteraceae coincide with marked shifts in transposable element abundance. BMC Genomics.

[17] Tetreault HM, Ungerer MC (2016). Long terminal repeat retrotransposon content in eight diploid sunflower species inferred from next-generation sequence data. G3 (Bethesda).

[18] Buti M, Giordani T, Cattonaro F, Cossu RM, Pistelli L, Vukich M, Morgante M, Cavallini A, Natali L (2011). Temporal dynamics in the evolution of the sunflower genome as revealed by sequencing and annotation of three large genomic regions. Theor Appl Genet.

[19] Staton SE, Bakken BH, Blackman BK, Chapman MA, Kane NC, Tang S, Ungerer MC, Knapp SJ, Rieseberg LH, Burke JM (2012). The sunflower (*Helianthus annuus* L.) genome reflects a recent history of biased accumulation of transposable elements. Plant J.

[20] Giordani T, Cavallini A, Natali L (2014). The repetitive component of the sunflower genome. Curr Plant Biol.

[21] Gill N, Buti M, Kane N, Bellec A, Helmstetter N, Berges H, Rieseberg LH (2014). Sequence-based analysis of structural organization and composition of the cultivated sunflower (*Helianthus annuus* L.) genome. Biology.

[22] Renaut S, Rowe HC, Ungerer MC, Rieseberg LH. Genomics of homoploid hybrid speciation: diversity and transcriptional activity of long terminal repeat retrotransposons in hybrid sunflowers. Philos T R Soc B. 2014;369(1648):20130345. 10.1098/rstb.2013.0345 PMC4071519 24958919

[23] Ungerer MC, Kawakami T (2013). Transcriptional dynamics of LTR retrotransposons in early generation and ancient sunflower hybrids. Genome Biol Evol.

[24] Sims LE, Price HJ (1985). Nuclear DNA content variation in *Helianthus* (Asteraceae). Am J Bot.

[25] Heiser CB, Smith DM, Clevenger SB, Martin WC (1969). The North American sunflowers (*Helianthus*). Mem Torrey Bot Club.

[26] Stephens JD, Rogers WL, Mason CM, Donovan LA, Malmberg RL (2015). Species tree estimation of diploid *Helianthus* (Asteraceae) using target enrichment. Am J Bot.

[27] Gevaert SD, Mandel JR, Burke JM, Donovan LA (2013). High genetic diversity and low population structure in porter's sunflower (*Helianthus porteri*). J Hered.

[28] Bino RJ, Lanteri S, Verhoeven HA, Kraak HL (1993). Flow cytometric determination of nuclear replication stages in seed tissues. Ann Bot-London.

[29] Novak P, Neumann P, Macas J (2010). Graph-based clustering and characterization of repetitive sequences in next-generation sequencing data. BMC Bioinformatics.

[30] Novak P, Neumann P, Pech J, Steinhaisl J, Macas J (2013). RepeatExplorer: a galaxy-based web server for genome-wide characterization of eukaryotic repetitive elements from next-generation sequence reads. Bioinformatics.

[31] Felsenstein J (1985). Phylogenies and the comparative method. Am Nat.

[32] Felsenstein J (1989). PHYLIP - phylogeny inference package (version 3.2). Cladistics.

[33] Tamura K, Stecher G, Peterson D, Filipski A, Kumar S (2013). MEGA6: molecular evolutionary genetics analysis version 6.0. Mol Biol Evol.

[34] Abascal F, Zardoya R, Posada D (2005). ProtTest: selection of best-fit models of protein evolution. Bioinformatics.

[35] Kelly LJ, Renny-Byfield S, Pellicer J, Macas J, Novak P, Neumann P, Lysak MA, Day PD, Berger M, Fay MF (2015). Analysis of the giant genomes of *Fritillaria* (Liliaceae) indicates that a lack of DNA removal characterizes extreme expansions in genome size. New Phytol.

[36] Natali L, Cossu RM, Barghini E, Giordani T, Buti M, Mascagni F, Morgante M, Gill N, Kane NC, Rieseberg L (2013). The repetitive component of the sunflower genome as shown by different procedures for assembling next generation sequencing reads. BMC Genomics.

[37] International Rice Genome Sequencing P (2005). The map-based sequence of the rice genome. Nature.

[38] Ming R, Hou S, Feng Y, Yu Q, Dionne-Laporte A, Saw JH, Senin P, Wang W, Ly BV, Lewis KL (2008). The draft genome of the transgenic tropical fruit tree papaya (*Carica papaya* Linnaeus). Nature.

[39] Paterson AH, Bowers JE, Bruggmann R, Dubchak I, Grimwood J, Gundlach H, Haberer G, Hellsten U, Mitros T, Poliakov A (2009). The Sorghum bicolor genome and the diversification of grasses. Nature.

[40] Argout X, Salse J, Aury JM, Guiltinan MJ, Droc G, Gouzy J, Allegre M, Chaparro C, Legavre T, Maximova SN (2011). The genome of Theobroma Cacao. Nat Genet.

[41] Gonzalez LG, Deyholos MK (2012). Identification, characterization and distribution of transposable elements in the flax (*Linum usitatissimum* L.) genome. BMC Genomics.

[42] Huang SW, Li RQ, Zhang ZH, Li L, Gu XF, Fan W, Lucas WJ, Wang XW, Xie BY, Ni PX (2009). The genome of the cucumber, *Cucumis sativus* L. Nat Genet.

[43] Jaillon O, Aury JM, Noel B, Policriti A, Clepet C, Casagrande A, Choisne N, Aubourg S, Vitulo N, Jubin C (2007). The grapevine genome sequence suggests ancestral hexaploidization in major angiosperm phyla. Nature.

[44] Buti M, Giordani T, Vukich M, Gentzbittel L, Pistelli L, Cattonaro F, Morgante M, Cavallini A, Natali L (2009). HACRE1, a recently inserted copia-like retrotransposon of sunflower (*Helianthus annuus* L.). Genome.

[45] Kawakami T, Dhakal P, Katterhenry AN, Heatherington CA, Ungerer MC (2011). Transposable element proliferation and genome expansion are rare in contemporary sunflower hybrid populations despite widespread transcriptional activity of LTR retrotransposons. Genome Biol Evol.

[46] Domingues DS, Cruz GMQ, Metcalfe CJ, Nogueira FTS, Vicentini R, Alves CD, Van Sluys MA. Analysis of plant LTR-retrotransposons at the fine-scale family level reveals individual molecular patterns. BMC Genomics. 2012;13:137. 10.1186/1471-2164-13-137 PMC3352295 22507400

[47] Ming R, VanBuren R, Wai CM, Tang HB, Schatz MC, Bowers JE, Lyons E, Wang ML, Chen J, Biggers E (2015). The pineapple genome and the evolution of CAM photosynthesis. Nat Genet.

[48] Vicient CM (2010). Transcriptional activity of transposable elements in maize. BMC Genomics.

[49] Meyers BC, Tingley SV, Morgante M (2001). Abundance, distribution, and transcriptional activity of repetitive elements in the maize genome. Genome Res.

[50] Schilling EE (1997). Phylogenetic analysis of *Helianthus* (Asteraceae) based on chloroplast DNA restriction site data. Theor Appl Genet.

[51] Bousios A, Gaut BS (2016). Mechanistic and evolutionary questions about epigenetic conflicts between transposable elements and their plant hosts. Curr Opin Plant Biol.

[52] Slotkin RK, Martienssen R (2007). Transposable elements and the epigenetic regulation of the genome. Nat Rev Genet.

[53] Lisch D (2009). Epigenetic regulation of transposable elements in plants. Annu Rev Plant Biol.

