# Supplementary material for: Genomic abundance and transcriptional activity of diverse gypsy and copia long terminal repeat retrotransposons in three wild sunflower species
Source: BMC Plant Biol. 2018 Jan 5;18:6. doi: 10.1186/s12870-017-1223-z (PMC5755311; doi:10.1186/s12870-017-1223-z)
Supplement: Additional file 1: Figure S1. — Pearson’s correlation between density of reads and nuclear genome size for gypsy and copia elements. Figure S2. Phylogenetic relationship among different sublineages of (A) gypsy and (B) copia based on amino acid sequences of the reverse transcriptase domain (gypsy, 173 a.a.; copia, 250 a.a.) using maximum likelihood. Bootstrap scores are presented for internal branches with >50% support. Table S1. Summary statistics of DNA-seq and RNA-seq data. (PDF 488 kb) [file 12870_2017_1223_MOESM1_ESM.pdf]

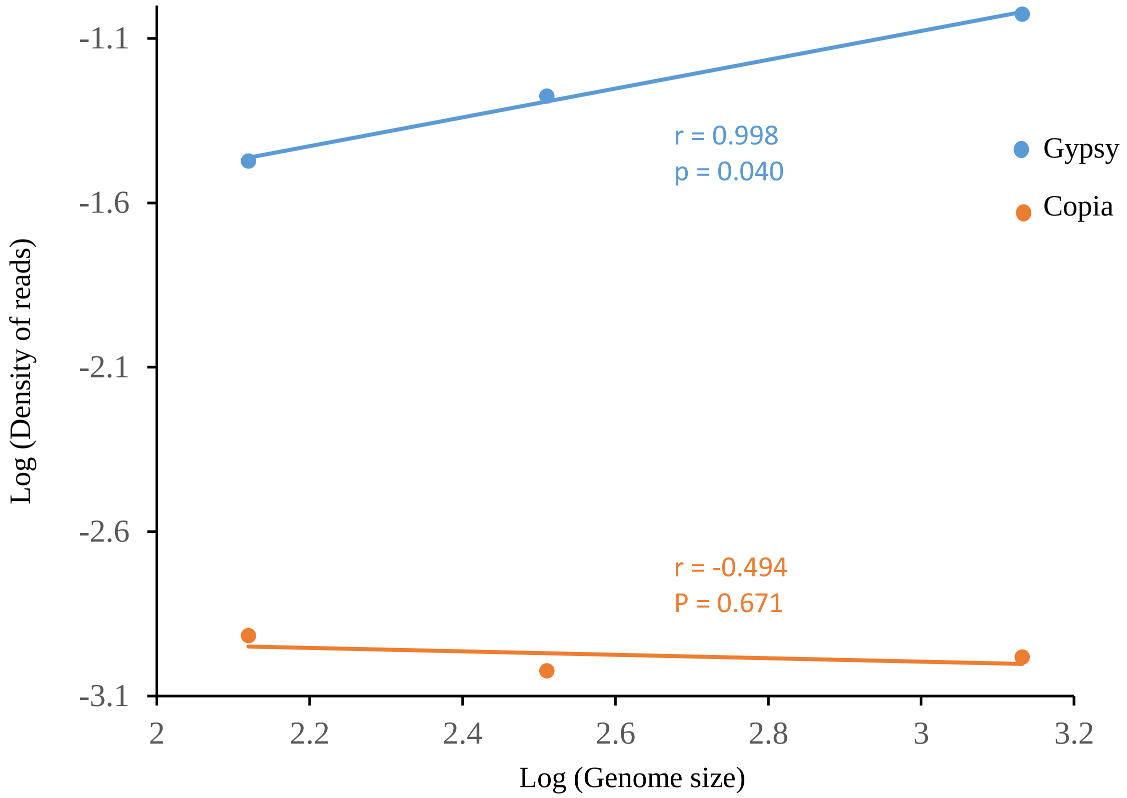

Figure S1 Pearson's correlation between density of reads and nuclear genome size for *gypsy* and *copia* elements.

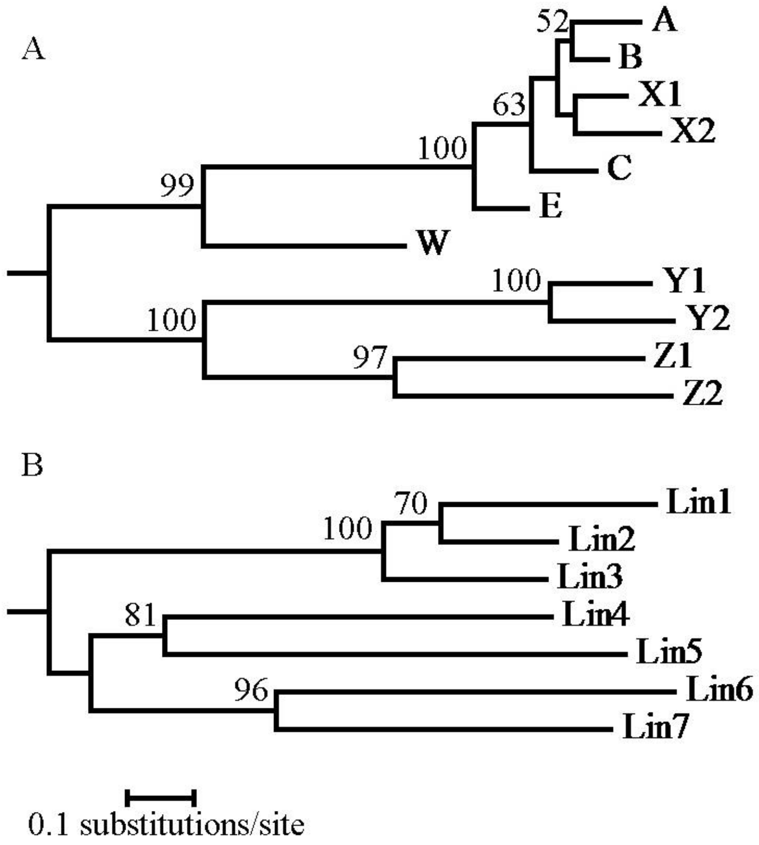

Figure S2 Phylogenetic relationship among different sublineages of (A) *gypsy* and (B) *copia* based on amino acid sequences of the reverse transcriptase domain (*gypsy*, 173 a.a.; *copia*, 250 a.a.) using maximum likelihood. Bootstrap scores are presented for internal branches with >50 % support.

Table S1 Summary statistics of DNA-seq and RNA-seq data

Sequencing type	Species	Replicates	Number of raw reads	Number of reads after trimming	Mean length of trimmed reads (bp)
DNA-seq	*H. agrestis*	AGR1	38,554,268	28,610,193	99.6
		AGR2	38,282,728	28,640,114	99.6
		AGR3	40,376,468	29,519,333	99.6
	H. carnosus	CAR1	34,000,952	24,639,844	99.6
		CAR2	36,392,088	26,776,432	99.6
		CAR3	38,609,046	28,317,411	99.6
	H. porteri	POR1	59,893,198	44,461,335	99.6
		POR2	63,510,716	48,040,581	99.7
		POR3	42,380,084	31,137,587	99.6
RNA-seq	*H. agrestis*	AGR1	39,956,738	36,263,031	93.7
		AGR2	43,991,344	39,901,894	93.8
		AGR3	41,116,568	37,346,480	93.9
	H. carnosus	CAR1	56,917,536	51,694,853	93.8
		CAR2	48,492,050	44,007,765	93.8
		CAR3	52,736,736	47,940,872	93.8
	H. porteri	POR1	44,362,868	40,207,966	93.8
		POR2	49,721,984	45,154,958	93.8
		POR3	35,754,786	32,591,321	93.8
